# Determinants of intimate partner controlling behavior targeting women in Türkiye

**DOI:** 10.3389/fpsyg.2023.1174143

**Published:** 2023-05-22

**Authors:** Burak Başkan, Ömer Alkan

**Affiliations:** ^1^Faculty of Economics and Administrative Sciences, Erzurum Technical University, Erzurum, Türkiye; ^2^Department of Econometrics, Faculty of Economics and Administrative Sciences, Atatürk University, Erzurum, Türkiye; ^3^Master Araştırma Eğitim ve Danışmanlık Hizmetleri Ltd. Şti., Ata Teknokent, Erzurum, Türkiye

**Keywords:** gender, intimate partner violence, controlling behavior, binary logistic regression, Türkiye

## Abstract

**Background/aim:**

Intimate partner controlling behavior toward women is an important form of intimate partner violence (IPV), both in terms of limiting women’s daily lives and in terms of reproducing patriarchal culture and male dominance in societies at the micro level. A limited number of studies in the literature have identified the male intimate partner’s controlling behavior as a dependent variable, which is important for understanding the determinants of this type of IPV. There is also a significant gap in the literature in terms of studies focusing on the case of Türkiye. Thus, the main aim of this study was to determine the socio-demographic, economic and violence-related factors that have an effect on women’s status in terms of exposure to control behavior in Türkiye.

**Methods:**

These factors were examined by using binary logistic regression analysis, based on the micro data set collected by the Hacettepe University’s Institute of Population Studies in the 2014-dated National Research on Domestic Violence against Women in Türkiye. A total of 7,462 women between the ages of 15 and 59 were interviewed face-to-face.

**Results:**

The findings of the study revealed that women are more likely to be exposed to controlling behavior if they live in rural areas, are unmarried, speak Turkish as their mother tongue, have bad or very bad health conditions, justify men’s violence and are afraid of their intimate partners. As women’s age, level of education and income contribution increase, their likelihood of exposure to controlling behavior decreases. However, women’s exposure to economic, physical and emotional violence also increases their likelihood of exposure to controlling behavior.

**Conclusion:**

The findings highlighted the importance of creating public policies that make women less vulnerable to men’s controlling behavior, providing women with methods and mechanisms of resistance and raising public awareness of the exacerbating effects of controlling behavior on social inequalities.

## 1. Introduction

Gender-based violence is one of the most significant human rights topics in today’s world. Based on the UN principle that any discrimination against women violates the equality of rights between men and women (UNGA, 1979), ‘gender-based violence’ has been categorized as “a form of discrimination that seriously inhibits women’s ability to enjoy rights and freedoms on a basis of equality with men” (CEDAW Committee, 1992) and the necessity to eliminate violence targeting women has been strongly emphasized by the World Conference on Human Rights in Vienna (UN, 1993). The most widespread form of gender-based violence is the ‘intimate partner violence’ (IPV) targeting women (Watts and Zimmerman, 2002; Devries et al., 2013; WHO, 2021). IPV corresponds to any kind of behavior within the boundaries of an intimate relationship that results in physical, sexual or psychological harm to the partner in the relationship. IPV comprises the acts of physical violence, emotional (psychological) abuse, sexual violence and controlling behaviors targeting the partner (WHO and PAHO, 2012). Acts of IPV can even result in intimate partner femicides. The number of girls and women murdered worldwide by their intimate partners or other family members was approximately 47,000 in 2020. A man in a relationship or a male family member murdered a girl or woman in every 11 min (UNODC, 2021).

IPV poses a severe threat to women’s physical health by causing high mortality rate among women, worse general level of health, injuries, chronic pain, disability, drug addiction, genital diseases and poor pregnancy experiences. IPV also leads to overuse of healthcare services, failure to fully meet healthcare needs, an increase in medical costs and distorted relationships with healthcare providers (Plichta, 2004). In addition to those cases in which women apply to primary healthcare providers for their immediate IPV-related injuries, women also experience lifelong health safety problems (Campbell et al., 2002). A correlation was determined between being exposed to IPV and experiencing mental disorders, namely depressive disorders, anxiety disorders and post-traumatic stress disorder (Trevillion et al., 2012). Moreover, significant studies reveal the effect of IPV on the prevalence of HIV in women (Jewkes et al., 2010; WHO and UNAIDS, 2010; UNAIDS, 2011). Exposure to gender-based violence, including a partner’s controlling behavior, may pave the way for highly risky sexual behaviors, including multiple and concurrent sexual partnerships, drug use, transgender sex and prostitution and less frequent use of condom. Women may accept riskier sex when they have been abandoned by their partners, are desperately seeking a relationship, are drugged, intoxicated or otherwise manipulated by controlling partners. In some cases, they may be less able to refuse. Thus, a vicious cycle of exposure to IPV, HIV infection and further exposure to IPV occurs (Jewkes et al., 2010).

Male intimate partner controlling behavior, on which this paper focuses, is categorized as a moderate form of IPV and is considered as an indicator of more damaging future violence acts (Aizpurua et al., 2021). The term refers to systematic efforts by one partner to control the movements and activities of the other partner and her social interactions with other people outside the home (Tun and Ostergren, 2020). The statement of ‘intimate partner controlling behavior’ in this paper comprises the controlling behavior of a former or current husband, a cohabiting partner or a dating partner targeting female partner in a heterosexual relationship. The main aim of this paper is to identify the socio-demographic, economic and violence-related factors that influence men’s controlling behavior toward women in Türkiye. Beyond being a purely empirical research, this paper also has a normative goal of contributing to gender equality in societies by highlighting the fact that male controlling behavior constructs structural barriers that prevent women from having the same conditions and opportunities as men.

The case of Türkiye, problematised in this paper, is significant to examine gender-based controlling behavior as it has a poor record in the fields of IPV and gender equality. Türkiye is among the countries with the highest lifetime IPV exposure rates in the world, with 32% of partnered or ever-married women aged 15–49 experiencing physical and/or sexual IPV (WHO, 2021). The country was ranked 124th out of 146 countries in the 2022 Global Gender Report in terms of gender equality (WEF, 2022). Women who had experienced controlling behavior among married women was detected as 77.6% in the country in 2004 (Mayda and Akkuş, 2005). The public health measures taken during the COVID-19 process such as isolation and mobility restrictions have also exacerbated male control over women with new means and opportunities to intervene in women’s life. In this period, 18.8% of women in Türkiye lost their job, while 46% of them started to devote less hours to working outside (UNWOMEN, 2020). These developments have caused women to be more vulnerable at home and have increased the extent of women’s isolation from social life, which are directly related to the issue of ‘intimate partner controlling behavior’. For these reasons, it is important to examine the factors that affect men’s controlling behavior toward women in the case of Türkiye.

## 2. Conceptual framework and literature review

Controlling behavior is one of the four different forms of IPV against women, along with sexual, physical and emotional (psychological) violence (Krug et al., 2002; WHO, 2005; Åsling-Monemi et al., 2008). It refers to isolation of a person from friends and family of birth; strict control over their movements and restrictions targeting one’s access to employment, education, medical care or financial resources (WHO and UNAIDS, 2010). In another definition, the term was defined as a range of acts aiming at positioning a person as subordinate and/or as dependent by depriving her/him of the means required for resisting, escaping and being independent; by exploiting her/his capabilities and resources for personal benefits; by ensuring her/his isolation from opportunities of support and by shaping her/his everyday life (CPS, 2017).

Although there are several studies that emphasize women’s controlling behaviors targeting men (Hines et al., 2007; Batinic et al., 2013; Walker et al., 2021; Waila et al., 2022); in most cases, women are in a position of victim, while men are in a position of perpetrator in a relationship of controlling behavior. Controlling behavior restricts a woman’s social and physical mobility, causes isolation and loss of autonomy and reduces her functional capacity in everyday life (Åsling-Monemi et al., 2008). Controlling behavior can be categorized as an invisible violence, as it is difficult to disclose. However, it may create psychologically more detrimental effects on victims, and cause more serious health problems than any other types of violence (Krantz and Vung, 2009). Controlling behavior corresponds to ongoing and frequent violence; while other types of violence that exclude controlling behavior generally arise from spontaneous fights, in which both sides may be aggressors (Durevall and Lindskog, 2015).

Men interiorise the attitude of control and domination over their partners through their experiences in a culture and learning from their families (Pence et al., 1993). More intimate partner violence (and accordingly controlling behavior) is experienced in those societies that have stronger ideologies constructed for ensuring male dominance. Those ideologies shape judicial systems, laws, attitudes of police, criminalisation of violence targeting women and consideration of women’s complaints regarding violence and abuses. Moreover, such ideologies have a significant limiting effect at societal level on issues such as women’s autonomy, their access to political circles, their impact on economy, their roles in the world of art and academia (Jewkes, 2002). These ideologies are the reflection of controlling behavior tendencies embedded within societies and by they are reproduced within societies by means of the daily controlling behavior practises in micro level.

Women also socialize in environments, which position them as subordinate, and injects men the idea of superiority to women. Thusly, the belief about men’s right to have a full control over women is a reflection of male patriarch phenomenon (Babu and Kar, 2009). In this environment, controlling behavior can result in gradual alteration of a woman’s views about herself, her relationship and her place in the world (Johnson and Ferraro, 2000). A significant outcome of this situation is women’s unconscious roles in justification of men’s tendency to control women and reproduction of patriarchal culture. Therefore, how controlling behavior is perceived varies across individuals and cultures, which complicates the measures that can be taken against controlling behavior. According to a study, controlling behaviors in a relationship can be regarded as irritating acts but not as abuse by adolescent women, therefore they do not cause the dissolution of the relationship (Baker and Carreño, 2016). Adolescent women justify jealousy and controlling behaviors of their partners by accepting them as a sign of true love (Williams, 2012). Thus, controlling behaviors become acts that are demanded rather than resisted, and reproduced in these relationships.

There are several types of behavior that have been identified in the literature as indicative of intimate partner controlling behavior. Trying to restrain a female partner from seeing or meeting her friends, restricting the opportunities of contact with her family of birth, persisting in knowing where she was at all times, getting angry if she contact or spoke with another man, being often suspicious that she was unfaithful (WHO, 2005; Åsling-Monemi et al., 2008; Krantz and Vung, 2009; Gilchrist et al., 2017; Aizpurua et al., 2021), demanding a female partner to ask permission before applying for healthcare for herself (WHO, 2005; Åsling-Monemi et al., 2008; Krantz and Vung, 2009; Gilchrist et al., 2017) and ignoring her and treating her indifferently (WHO, 2005; Åsling-Monemi et al., 2008; Gilchrist et al., 2017) are included within the scope of controlling behavior by significant studies in the literature. Forbidding female partner to wear some types of clothes (Chacham et al., 2016) and taking control over what to wear (CPS, 2017) were also regarded as controlling behaviors in prominent researches. Thus, a woman’s appearance is determined according to the preferences of men, and a woman’s right to express her identity and character through her appearance is restricted by men.

Intervening in partner’s use of social networks such as Facebook, Instagram and Twitter can also be regarded as a new indicator of controlling behavior in relationships. The technology usage in communication and especially the use of social media increases controlling behavior in relationships. First, it facilitates the means of monitoring the partner (Belotti et al., 2022). The Internet environment decreases relational uncertainty while simultaneously increasing controlling behavior, as the partner can easily monitor his partner’s wall postings, friend lists, event invitations, photos and status updates (Ruggieri et al., 2021). It triggers jealousy, since communication with other potential partners on social media is much easier (Baker and Carreño, 2016). Deleting contacts or friends from a partner’s online accounts or mobile phone and preventing a partner from using the electronic communication technology in order to restrain her from talking to others can be two controlling partner behavior to isolate and control women in everyday life (Stonard, 2019). Within this scope, Daspe et al. (2018) detected a significant relationship between Facebook use, Facebook jealousy and exposure to IPV.

The meaning and forms of behavior covered by the term ‘controlling behavior’ have varied over time and similar terms with close or complementary meanings have emerged in the literature. Johnson (1995) positioned the motivation for controlling the partner as the main determiner of differentiation between patriarchal terrorism (or intimate terrorism) and common couple violence (or situational couple violence). He introduced intimate terrorism as a general strategy of control and power over the partner. However, he argued that situational couple violence does not involve violence and cannot be regarded as a general control pattern, since it is presumably an outcome of the exacerbation of couple conflict toward violence (Johnson, 2006). Stark (2007) conceptualized the term ‘coercive control’ by emphasizing its fundamental motivation aiming to target women’s gender-based vulnerability and to ensure men’s privileged position in terms of having control on resources, daily life and usage of time. Kelly and Johnson (2008) used the term ‘coercive controlling violence’ as a mutated version of controlling behavior by defining it as a punitive action that targets the subordinate side within a relationship in case of failing to obey the rule set by coercive partner. Within the frame of the Domestic Abuse Intervention Project’s findings based on the instances of controlling behaviors continually notified by both perpetrators and victims (Pence et al., 1993), controlling behavior was regarded as an extended term by Graham-Kevan and Archer (2003, 2008) so that it involved economic measures, threats, intimidation, emotional abuse and isolation. Thus, this scale went beyond the restriction and isolation of women from everyday life and introduced a broader understanding of control that included indirect factors that determine women’s autonomy in everyday life.

Several population-based surveys aiming at measuring controlling behavior have been carried out in different countries around the world. Controlling behavior rate was detected as 64% in England, while it is detected as 65% in Brazil (Gilchrist et al., 2017). The proportion of women exposed to one or more controlling behavior acts was 21% in the urban areas of Japan, while it was 90% in the urban areas of the United Republic of Tanzania (Garcia-Moreno et al., 2006). In Kano city of Nigeria, harassment or controlling behavior was found as the most common form of male violence with 43.3% (Tanimu et al., 2016), while it was 31.5% in Lagos, Nigeria (Igwe et al., 2021). In Pakistan, 31.8% of women aged between 15 and 24 years reported that they had been exposed to controlling behavior of their husbands (Nasrullah et al., 2014). In Myanmar, 30.2% of women have been determined as being exposed to controlling behavior (Tun and Ostergren, 2020), while it was 39.7% for women in North Kerala, India (Mundodan et al., 2021).

Significant studies detected the effects of controlling behavior on other forms of IPV (physical, sexual and emotional or psychological violence) (Bradley et al., 2002; Kishor and Johnson, 2005; WHO, 2005; Graham-Kevan and Archer, 2008; Antai, 2011; Fawson, 2015; Aizpurua et al., 2021; Kanougiya et al., 2021; Mukherjee and Joshi, 2021; McClintock et al., 2022). The original contribution of the current study, on the other hand, stems from its repositioning controlling behavior as the dependent variable while endeavoring to measure the effects of other violence types on controlling behavior against women. From this point of view, the physical forms of IPV (namely, physical and sexual violence) and controlling behavior are inseparable as they mostly foreshadow one another. As one partner dominates a relationship by using physical or sexual violence, the risk of perpetual violence increases since the dominant partner seeks to sustain the domination or the subordinate partner seeks to challenge the restrictions imposed by the dominant partner, or to reverse the current power structure within the relationship (Straus, 2008). The violence of a man, in this case, is instrumentalised for demonstrating and enforcing his position as head of the relationship or household (Watts and Zimmerman, 2002) and motivated by his desire to assure general control over “his” woman (Johnson, 1995). Appeal to physical or sexual assault, on the other hand, strengthens dominant partner’s capacity and power to use other nonphysical control tactics such as isolation (and thus, controlling behavior), threat and emotional abuse, which results in the suppression of subordinate partner’s ability for autonomous action (Pence et al., 1993).

## 3. Methods

### 3.1. Study design and sample

The current study is a secondary data analysis, in which cross-sectional data (human data) gathered by the Hacettepe University’s Institute of Population Studies in the 2014-dated National Research on Domestic Violence against Women in Türkiye have been used. The research carried out nationwide for the first time in 2008 has become a prominent study in the field of gender inequality for it provided comprehensive data for the first time to understand different aspects of violence against women (DGSW, 2009). The 2014-dated version of the same research has improved the contribution of 2008-dated research by revealing the change in terms of gender-based violence between 2008 and 2014 (DGSW, 2015). The questionnaire used in both researches were adapted from the “Multi-Country Study on Women’s Health and Domestic Violence against Women” conducted by the World Health Organization (WHO, 2005). Considering technological advances since 2005 and the peculiarities of Türkiye, however, questionnaire became more comprehensive with newly added questions. The research, which investigated women between 15 and 59 years old, was carried out between April 8, 2014 and July 11, 2014 (DGSW, 2015).

### 3.2. Setting

Within the scope of the National Research on Domestic Violence against Women in Türkiye, the country was stratified into 30 layers, by which Türkiye as a whole with its urban and rural areas could be reflected. Regional classifications were made under two categories: 12 regions as İstanbul, Aegean, West Marmara, East Marmara, Mediterranean, West Anatolia, Central Anatolia, Central East Anatolia, East Black Sea, Northeast Anatolia, West Black Sea and Southeast Anatolia and 5 regions as West, East, Central, North and South. In each region, urban strata constituted 75%, while rural strata accounted for 25%. The only exception was Istanbul, where only about 5% of the households were rural. In the study, settlements in which less than 10,000 people dwelled were accepted as rural strata, while settlements in which 10,000 or more people dwelled were accepted as urban strata (DGSW, 2015).

The process of sample selection was achieved by the collaboration of the Turkish Statistical Institute (TURKSTAT). The number of women found in the interviewed households was 13,310 who were aged between 15 and 59. After collecting the data about their age, education and marital status, 8,960 of them were determined as potential eligible interviewees by applying the Kish method. Face-to-face interviews were completed with 7,462 of those women, which corresponded to a response rate of 83.3%. Only 4.4% of them refused to respond to the questions (DGSW, 2015).

### 3.3. Bias

Since the data about women’s experiences of exposure to men’s controlling behavior were based on the subjective responses of women, the research had a risk of comprising biased data as other similar researches using this method have.

### 3.4. Limitations of the study

The study has some limitations. Firstly, the study relies on secondary data. As the variables used in the statistical analysis are the variables included in the dataset, this study could not go beyond the dataset in question in terms of measuring different variables. Moreover, another limitation is the lack of questions to measure how IPV in general and controlling behavior in particular affect women’s spirituality.

### 3.5. Ethical and safety procedures

Field team members were selected from among university graduates or students under the age of 30 who were able to work continuously during the field study process. A two-week training program was carried out for these team members. The training program focused on topics such as interviewing techniques, typical questionnaire applications, domestic violence and gender issues. The training was conducted by the project assistants and academic staff of HUIPS, and experts from different organizations and institutions. The program contributed to raise the awareness of interviewers and provided them with techniques to gather information without disturbing the interviewed women (DGSW, 2015).

Each stage of the study was guided by ethical guidelines prepared by the WHO to assure the safety of both the interviewer and the women interviewed. The title of the study was determined so as not to include the word violence in order to avoid further violence against the women interviewed, and the details of the study were not shared with anyone other than these women. Questions were asked after obtaining the respondent’s consent, and only one woman from each household was interviewed to avoid over-representation of any household. Field researchers were trained in the confidentiality of the research and supervised by research supervisors and academic staff. Non-governmental organizations and public institutions dealing with domestic violence were informed about the women who said they were exposed to any form of violence in order to ensure their safety (DGSW, 2015).

### 3.6. Dependent variables

Within the frame of the National Research on Domestic Violence against Women in Türkiye, women were asked the several behaviors of their intimate partners that targeted them. These behaviors comprise ‘Preventing the woman from seeing her friends’, ‘Preventing the woman from seeing her own family and relatives’ (The phrase ‘her own family’ refers to a woman’s family of birth), ‘Always wanting to know the whereabouts of the woman’, ‘ignoring the woman and treating her indifferently’, ‘getting angry when the woman talks to other men’, ‘Suspecting that the woman is unfaithful’, ‘Demanding the woman to ask for his permission to go to a health institution’ ‘Interfering with the woman’s clothing and demanding her to dress as he wants’ and ‘Interfering with the woman’s use of social networks such as Facebook and Twitter’ (DGSW, 2015).

Those questions were responded by the participant women as ‘yes’ or ‘no’ and women’s experience of controlling behavior measured by these questions created the dependent variable of the current study. Exposure to controlling behavior was coded as ‘1’ if the respondent woman had experienced at least one of those nine behavior types, and as ‘0’ if she had not experienced any of them.

### 3.7. Independent variables

This current study based its independent variables on sociodemographic and economic characteristics of participants and on matters regarding different types of IPV’s (economic, physical and emotional violence) other than controlling behavior. These independent variables are sourced from the National Research on Domestic Violence against Women in Türkiye. The variables regarding participants’ sociodemographic characteristics and economic conditions were the place of residence (rural or urban), age (15–24, 25–34, 35–44, 45–54 or 55+), mother tongue (Turkish or another language such as Kurdish, Arabic etc.), marital status (married or unmarried), health status (bad/very bad, reasonable or excellent/good), educational level of the participant woman (illiterate/have no diploma, elementary school, secondary school, high school and university), educational level of participants’ intimate partner (illiterate/have no diploma, elementary school, secondary school, high school and university), household income level (1st income level, 2nd income level, 3rd income level and 4th income level) and woman’s status of having a higher income contribution to the household (no, yes).

Violence-related variables in the study were women’s experience of exposure to economic violence from the intimate partner at any time in their lives (no, yes), intimate partner’s experience of fight with another man that results in physical violence (no, yes), women’s experience of exposure to physical violence from the intimate partner at any time in their lives (no, yes), women’s finding it right for the men to beat their partners or wives (no, yes), women’s experience of exposure to emotional violence from the intimate partner at any time in their lives (no, yes), women’s experience of exposure to emotional violence from someone other than the intimate partner at any time in their lives (no, yes) and women’s situation of being afraid of the intimate partner (no, yes).

### 3.8. Statistical analysis

STATA version 15, which was released in 2017, was preferred for this study. Survey statistics in STATA 15 (Stata Corporation) were used to reckon with weights and the complex sampling design. At this stage, weighted analysis was carried out (Alkan et al., 2022). Frequencies and percentages were obtained according to women’s exposure to controlling behavior. Chi-square independence test was conducted to examine the relationship between the exposure to controlling behavior and independent variables. Risk factors that are associated with exposure to controlling behavior were then identified using binary logistic regression analysis.

## 4. Results

### 4.1. Descriptive statistics and chi-square test

Table 1 reveals how sociodemographic and economic factors as well as different types of IPV (physical violence, economic violence and emotional violence) affect men’s controlling behavior against women. 81.7% of all women participating in the study declared that they have been exposed to one of the 9 behavioral patterns that are considered as controlling behavior symptoms at least once in their lives.

**Table 1:** Findings about the factors that affect women’s status of being exposed to controlling behavior.

Variables	Controlling behavior experience		*n* (%)	*χ*^2^	*p*
	No	Yes			
Place of residence					
Urban Area	864 (13.4)	3,540 (54.8)	4,404 (68.2)	17.215	0.000
Rural Area	315 (4.9)	1,739 (26.9)	2,054 (31.8)		
Age					
15–24	85 (7.2)	718 (13.6)	803 (12.4)	84.063	0.000
25–34	291 (24.7)	1,694 (32.1)	1,985 (30.7)		
35–44	363 (30.8)	1,387 (26.3)	1,750 (27.1)		
45–54	324 (27.5)	1,088 (20.6)	1,412 (21.9)		
55+	116 (9.8)	392 (7.4)	508 (7.9)		
Mother tongue					
Turkish	1,003 (85.1)	4,286 (81.2)	5,289 (81.9)	9.799	0.002
Kurdish, Arabic etc.	176 (14.9)	993 (18.8)	1,169 (18.1)		
Marital status					
Married	1,059 (89.8)	4,495 (85.1)	5,554 (86.0)	17.483	0.000
Unmarried	120 (10.2)	784 (14.9)	904 (14.0)		
Health status					
Excellent/Good	612 (51.9)	2,345 (44.4)	2,957 (45.8)	23.908	0,000
Reasonable	444 (37.7)	2,209 (41.8)	2,653 (41.1)		
Bad/Very Bad	123 (10.4)	725 (13.7)	848 (13.1)		
Educational level					
Illiterate/Have No Diploma	165 (14.0)	1,076 (20.4)	1,241 (19.2)	79.988	0.000
Elementary School	525 (44.5)	2,382 (45.1)	2,907 (45.0)		
Secondary School	134 (11.4)	745 (14.1)	879 (13.6)		
High School	202 (17.1)	714 (13.5)	916 (14.2)		
University	153 (13.0)	362 (6.9)	515 (8.0)		
Educational level of the intimate partner					
Illiterate/Have No Diploma	55 (4.7)	288 (5.5)	343 (5.3)	51.879	0.000
Elementary School	430 (36.5)	2,321 (44.0)	2,751 (42.6)		
Secondary School	190 (16.1)	873 (16.5)	1,063 (16.5)		
High School	283 (24.0)	1,179 (22.3)	1,462 (22.6)		
University	221 (18.7)	618 (11.7)	839 (13.0)		
Household income level					
1. Income Level	251 (21.3)	1,387 (26.3)	1,638 (25.4)	17.663	0.000
2. Income Level	282 (23.9)	1,309 (24.8)	1,591 (24.6)		
3. Income Level	312 (26.5)	1,310 (24.8)	1,622 (25.1)		
4. Income Level	334 (28.3)	1,273 (24.1)	1,607 (24.9)		
Women having a higher income contribution to the household					
Yes	68 (5.8)	254 (4.8)	322 (5.0)	1.860	0.183
No	1,111 (94.2)	5,025 (95.2)	6,136 (95.0)		
Exposure to economic violence from the intimate partner					
Yes	147 (12.5)	1,607 (30.4)	1,754 (27.2)	157.368	0.000
No	1,032 (87.5)	3,672 (69.6)	4,704 (72.8)		
Intimate partner’s fight with another man that results in physical violence					
Yes	52 (4.4)	603 (11.4)	655 (10.1)	51.996	0.000
No	1,127 (95.6)	4,676 (88.6)	5,803 (89.9)		
Exposure to physical violence from the intimate partner					
Yes	187 (15.9)	1,942 (36.8)	2,129 (33.0)	190.980	0.000
No	992 (84.1)	3,337 (63.2)	4,329 (67.0)		
Women’s finding it right for the men to beat their partners/wives					
Yes	261 (22.1)	2,257 (42.8)	2,518 (39.0)	172.210	0,000
No	918 (77.9)	3,022 (57.2)	3,940 (61.0)		
Exposure to emotional violence from the intimate partner					
Yes	265 (22.5)	2,398 (45.4)	2,663 (41.2)	209.456	0.000
No	914 (77.5)	2,881 (54.6)	3,795 (58.8)		
Exposure to emotional violence from someone other than the intimate partner					
Yes	162 (13.7)	1,159 (22.0)	1,321 (20.5)	39.968	0.000
No	1,017 (86.3)	4,120 (78.0)	5,137 (79.5)		
Being afraid of the intimate partner					
Yes	84 (7.1)	986 (18.7)	1,070 (16.6)	93.057	0.000
No	1,095 (92.9)	4,293 (81.3)	5,388 (83.4)		

68.2% of women participated in the research are from urban areas of Türkiye, while 31.8% of them are from rural areas of the country. Women from 25 to 34 years old constituted the largest group of participants in terms of age with the percentage of 30.7%. Women between 35 and 44 and women between 45 and 54 followed them with the percentages of 27.1 and 21.9%, respectively. The percentage of women whose mother tongue is Turkish among all researchers is 81.9%. 86% of women participated in the research are married, while 14% of them are unmarried. While 41.1% of women participated in the research have reasonable health status, 13.1% of them have bad or very bad health status.

Women who are elementary school graduates constituted the largest percentage of research participants with 45%. They were followed by the illiterate women with 19.2%. Moreover, 45% of participants’ intimate partners are primary school graduates, while 22.6% of them are high school graduates and 16.5% of them are secondary school graduates. The fact that only 5.3% of men are illiterate as compared to 19.2% of women’s illiteracy reveals important results about the unequal distribution of power within families. 25.4% of participant women were from first income group, while 24.6% of them were from second income group, 25.1 from third income group and 24.9% from fourth income group. Although the proportion of women who contribute more to household income than their intimate partners is 5% in the research, it provides important insights about the relationship between women’s financial independence and their capacity to overcome men’s controlling behavior.

According to the responses of the participants of the research, 33% of women expressed that they were exposed to physical violence, 41.2% to emotional violence and 27.2% to economic violence. In the research, 10.1% of women declared that their intimate partner had experienced a fight with another man leading to physical violence. 39% women declared that they found it right for the men to beat their partners or wives. 20.5% of them stated that they had been exposed to emotional violence from someone other than their intimate partners. 18.7% of participants declared that they were afraid of their intimate partners. Thus, the research comprises a wide range of socio-demographic and violence-related factors that affect women’s status of being exposed to male controlling behavior.

### 4.2. Estimation of models

Binary logistic regression model has been used in order to comprehend the factors that affect women’s status of being exposed to controlling behavior. The results regarding the estimated model have been presented in Table 2. In the research, the variables about woman’s place of residence, woman’s age, woman’s mother tongue, marital status, woman’s health status (excellent/good), woman’s educational level (high school and university), educational level of the intimate partner (illiterate/have no diploma), household income level (2. income level), woman’s higher income contribution to the household, woman’s exposure to economic violence from her intimate partner, intimate partner’s fight with a man that results in physical violence, woman’s finding it right for the men to beat their partners or wives, woman’s exposure to emotional violence from her intimate partner, exposure to physical violence from her intimate partner, woman’s status of being afraid of her intimate partner and women’s exposure to emotional violence from someone other than the intimate partner have been detected as significant.

**Table 2:** The results of estimated binary logistic regression model and marginal effects about the factors that affect women’s status of being exposed to controlling behavior.

Variables	*β*	Std. error	95% CI		ME (%)	Std. error	VIF
			Lower	Upper			
Place of residence (Reference: Rural Area)							
Urban Area	−0.191	0.093	−0.374	−0.009	−3.5^b^	0.016	1.16
Age (Reference: 15–24)							
25–34	−0.350	0.181	−0.706	0.004	−4.3^c^	0.020	3.12
35–44	−0.957	0.184	−1.319	−0.595	−15.4^a^	0.025	3.24
45–54	−1.158	0.192	−1.536	−0.781	−20.2^a^	0.029	3.15
55+	−1.322	0.226	−1.766	−0.877	−24.6^a^	0.045	2.01
Mother tongue (Reference: 15–24)							
Turkish	0.216	0.124	−0.027	0.460	4.3^c^	0.026	1.34
Marital status (Reference: Unmarried)							
Married	−0.444	0.143	−0.725	−0.163	−7.5^a^	0.021	1.36
Health status (Reference: Bad/Very Bad)							
Excellent/Good	−0.244	0.144	−0.526	0.038	−4.5^c^	0.025	2.87
Reasonable	−0.110	0.140	−0.386	0.164	−1.9	0.024	2.55
Educational level (Reference: Illiterate/Have No Diploma)							
Elementary School	−0.202	0.135	−0.467	0.062	−3.4	0.021	2.42
Secondary School	−0.310	0.189	−0.681	0.061	−5.4	0.033	2.18
High School	−0.420	0.182	−0.778	−0.063	−7.7^b^	0.033	2.34
University	−0.648	0.210	−1.060	−0.236	−12.9^a^	0.043	2.28
Educational level of the intimate partner (Reference: Elementary School)							
Illiterate/Have No Diploma	−0.394	0.200	−0.786	−0.001	−8.1^b^	0.046	1.19
Secondary School	−0.183	0.128	−0.436	0.068	−3.5	0.025	1.32
High School	−0.151	0.119	−0.385	0.082	−2.8	0.022	1.50
University	−0.063	0.146	−0.351	0.224	−1.1	0.027	1.84
Household income level (Reference: 1. Income Level)							
2. Income Level	0.221	0.123	−0.020	0.463	4.4^c^	0.025	1.66
3. Income Level	0.184	0.125	−0.061	0.430	3.7	0.025	1.72
4. Income Level	0.200	0.126	−0.046	0.447	4.0	0.025	1.80
Women having a higher income contribution to the household (Reference: No)							
Yes	−0.397	0.174	−0.739	−0.054	−8.6^b^	0.042	1.17
Exposure to Economic Violence from the Intimate Partner (Reference: No)							
Yes	0.815	0.117	0.584	1.046	13.1^a^	0.015	1.17
Intimate partner’s fight with another man that results in physical violence (Reference: No)							
Yes	0.412	0.183	0.053	0.772	6.9^b^	0.026	1.10
Exposure to Physical Violence from the Intimate Partner (Reference: No)							
Yes	0.525	0.115	0.300	0.751	9.1^a^	0.018	1.50
Women’s finding it right for the men to beat their partners/wives (Reference: No)							
Yes	0.799	0.097	0.608	0.991	13.6^a^	0.015	1.13
Exposure to Emotional Violence from the Intimate Partner (Reference: No)							
Yes	0.472	0.102	0.272	0.673	8.5^a^	0.017	1.45
Exposure to emotional violence from someone other than the intimate partner (Reference: No)							
Yes	0.196	0.114	−0.028	0.422	3.6^c^	0.020	1.10
Being afraid of the intimate partner (Reference: No)							
Yes	0.480	0.147	0.190	0.770	8.0^a^	0.021	1.17
Constant	2.109	0.252	1.613	2.604	Mean VIF:		1.82

^a^*p* < 0.01; ^b^*p* < 0.05; ^c^*p* < 0.10. VIF, Variance Inflation Factor.

Based on the binary logistic regression model introduced in Table 2, while holding other variables constant, women living in urban areas of Türkiye have lower possibility of exposure to intimate partner controlling behavior by 3.5% as compared to women living in rural areas. As compared to those women between 15and 24 years old, women who are between 25 and 35 years old possessed a less possibility of exposure to controlling behavior by 4.3%, women between 35 and 44 years old by %15.4, women between 45 and 54 years old by 20.2% and women over 55 years old by 24.6%. Women who speaks Turkish as a mother tongue had more possibility of being exposed to controlling behavior by 4.3% *vis-à-vis* those women whose mother tongue is different from Turkish. Married women are 7.5% less likely to experience controlling behavior than unmarried women. Women with excellent or good health status have less possibility of being exposed to controlling behavior by 4.5% compared to those women with bad or very bad health status.

Women who are a high school graduate possessed a less possibility of exposure to their intimate partner’s controlling behavior by 7.7% and women who are a university graduate by 12.9%. Women whose intimate partners are illiterate (or have no diploma) are 8.1% less likely to face with controlling behavior than women whose intimate partners had graduated from elementary school. Women with second income level possess higher possibility of exposure to controlling behavior compared to those from with first income level. Women having a higher income contribution to the household are less likely to be exposed to controlling behavior than other women are by 8.6%.

Table 2 also reveals the effects of variables about effects of other types of IPV on controlling behavior. Women who have been exposed to economic violence in the past have more possibility of being exposed to controlling behavior by 13.1% compared to women who had not been exposed to economic violence in their lifetime. When intimate partners fight with another man ending up with physical violence, women’s probability of exposure to controlling behavior of their intimate partners increases by 6.9%. The possibility of being exposed to controlling behavior is 9.1% higher for women who have experienced physical violence from their intimate partners as compared to those women have not experienced physical violence in their life. Women who find it right for the men to beat their partner or wife are more likely to be exposed to controlling behavior by 13.6% than women who find it wrong. Women who experienced emotional violence from their intimate partners are 8.5% more likely to be exposed to controlling behavior. Women who experienced emotional violence from someone other in their lifetime than their intimate partners are 3.6% more likely to be exposed to controlling behavior from their intimate partners. Women who are afraid of their intimate partners possess 8% more possibility of being exposed to controlling behavior.

## 5. Discussion

Male controlling behavior targeting women is a form of male dominance over female, deeply rooted in historical unequal gender norms of patriarchy in societies. Male-dominated social setting promotes the idea of men’s superiority and endows men the right to have a control over women (Mondal and Paul, 2021). Patriarchal social and cultural structure in Türkiye also reproduces a social setting that encourages men’s controlling behaviors over women (Algül and Yarbaşı, 2021).

A possible reason for a higher possibility of exposure to controlling behavior in rural areas might be the patriarchal culture prevailing in those areas that is mostly supported by strong traditions and social bonds that impose those traditions. Rural life is characterized by strict social control established by the rural communities. Women’s empowerment within their families becomes impossible due to inescapable close relations with peers and relatives in villages (Erman, 2001). A woman can only find opportunities of realizing her potential when she moves to a city with her husband and possesses a nuclear family far from the extended family of her husband (Erman, 1998). In isolated rural areas, moreover, women possess less opportunity of accession to social services (Velzeboer et al., 2003), while they have different opportunities to access to TV, newspapers, Internet and other similar media tools more easily which help them to be aware of their legal rights in urban areas (Alkan and Tekmanlı, 2021). Another study conducted in East Timor, however, reached an opposite result by indicating that women living in urban areas possess higher probability of being exposed to controlling behavior as compared to those women living in rural areas (NSD, 2010).

Based on the findings of the current research, the possibility of being exposed to controlling behavior decreases as the age of women increases. The study conducted in Ankara province of Türkiye determined that the frequency of exposure to controlling behavior was determined as 64.3% for the ages between 25 and 34, while it was determined as 50.6% for the ages between 15 and 24 (Akar et al., 2010). In urban Karnataka city of India, the likelihood of women being exposed to controlling behavior steadily decreases for women after 30 years old (Kundapur et al., 2017). Similarly, younger women more commonly experience controlling behaviors according to the study conducted in Spain (Aizpurua et al., 2021). As Farmer and Tiefenthaler (1996) underlined, as men feel a credible threat of leave by women, women can have a better control over violence and controlling behaviors. Since young women can have more opportunities outside the relationship, men will feel more threat in their relationship or marriage with young women. This will lead to a tighter control of men over younger women.

Considering the mother tongue of the participants, which is directly related with the ethnicity, Turkish-speaking women are more likely to be exposed to controlling behavior as compared to women whose mother tongue is different from Turkish. Ozer and Fidrmuc (2017) found that being Kurdish did not have a significant effect on controlling behavior of men, while being from the other ethnic origins other than Turkish and Kurdish lowered the possibility of exposure to controlling behavior.

According to the current research, married women are less likely to be exposed to controlling behavior as compared to unmarried women. A possible reason for such a finding may be that men regard their dominance over women as more guaranteed when they are married. However, an important reason why unmarried women are exposed to controlling behavior by their partners is that men expect the security of the partnership provided by the marriage bond for married couples from controlling behavior. On the other hand, marriage does not guarantee a life without male controlling behavior for women. Marriage in rural areas, especially in Eastern Türkiye, poses a significant obstacle against women’s autonomy, since it is expected from the bride that she should live as a subordinate with her husband’s extended family (Erman, 2001). An empirical study conducted in Spain also determined that women in marital relationships are less likely to experience controlling behaviors from their husbands (Aizpurua et al., 2021).

Based on the findings regarding the health status participant women, having an excellent or good health status decreases the possibility of exposure to controlling behavior as compared to having bad or very bad health status. Similarly, Fanslow et al. (2021) detected a positive correlation between women’s health disabilities and their exposure to controlling behavior by men in the case of New Zealand. A possible reason for such a result may be that women having bad or very bad health status are not able to develop resistance against men’s domination due to their physical or psychological health disabilities.

According to the findings about the educational level, women who are high school or university graduates are less likely to be exposed to controlling behavior as compared to those women who are illiterate or have no diploma. In another study focused on the case of Ankara province of Türkiye, it was also determined that women’s possibility of exposure to controlling behavior increases, as they possess a lower level of education (Akar et al., 2010). Studies conducted in India found that women with lower educational status are more likely to be exposed to controlling behavior (Ackerson et al., 2008; Mukherjee and Joshi, 2021).

The findings of the current research about the educational level of the intimate partner showed that women are less likely to be exposed to controlling behavior when their intimate partners are illiterate or have no diploma as compared to those women whose intimate partners are elementary school graduates. In the research, however, the correlation between the educational level of intimate partner when he is a secondary school, high school or university graduate and women’s exposure to controlling behavior is not significant. Ozer and Fidrmuc (2017) determined that the correlation between husband’s education level and his attitude of controlling behavior against his wife was not statistically significant. On the other hand, Martin et al. (2002) reached the result in the case of India that men with less than 6 years of education are more likely to resort to controlling behaviors toward their wives.

The current research found that women from the first household income level are less likely to be exposed to controlling behavior as compared to those women from second household income level. According to a study conducted in East Timor, women with the highest level of household income level possess the highest possibility of exposure to controlling behaviors of their husbands (NSD, 2010). Akar et al. (2010), however, detected that women are more likely to be exposed to controlling behavior as the household income level becomes lower. For the men from lower income level, in this situation, violence can be regarded as an alternative resource to economic resources in order to control others (and especially their wives and children) for the service of their ends and to achieve their goals (Goode, 1971).

The current research determined that those women who have a higher income contribution to their household are less likely to be exposed to controlling behavior by their intimate partners. Erten and Keskin (2018) found a different result by indicating that a higher income of women by means of better schooling incites men to resort to controlling behavior and to threat of violence in order to ensure their control over the decision-making of the household resource allocation. This counterargument is supported by another argument that the incomes of most women are directly transferred to the household’s common revenue fund (Bobonis et al., 2013). On the other hand, narrowing the male–female wage gap between male and female increases women’s bargaining power within the family (Aizer, 2010). When women possess more opportunities outside the home with an increasing income and various job options, their threat to leave the family becomes more credible and women can have more effect on the distribution of resources within the family (Hidrobo and Fernald, 2013). In the Nigerian context, when women have more financial autonomy, they are almost twice as likely to negotiate safer sex than women who have no financial autonomy (Solanke et al., 2023), which is directly linked to possessing ability to avoid exposure to controlling behavior.

According to the findings of the current research, exposure to economic violence significantly rises the risk of being exposed to controlling behavior. Economic violence is often used interchangeably with the terms of ‘economic control’ or ‘financial control’ and manifests itself as a controlling behavior that unreasonably hinders a person’s financial autonomy (Macdonald, 2012). It includes those controlling acts against a person’s access to money, clothes, food and personal belongings such as bankbook or car keys or baseless denial of the instruments required for attendance to social life (ABS, 2009). Therefore, economic violence can be regarded as a reflection of the tendency to control and stems from the same motivation as controlling behavior. The studies in the literature tend to evaluate economic violence as a part of controlling behavior (Graham-Kevan and Archer, 2003, 2008; Ali et al., 2014), therefore the current study contributes to the literature by approaching the two concepts separately and investigating the correlation between each other.

According to the findings of the current research, when a woman’s intimate partner’s fight with another man results in physical violence, she is more likely to be exposed to controlling behavior. Since such a result reveals a man’s predisposition to fight leading physical violence, it is also understandable that a woman is more likely to be subjected to controlling behavior by this man. No other study examining the correlation between these two variables has been found in the literature.

The current study also found that women were more likely to be exposed to controlling behavior, when they had experienced physical violence in the past. In the case of Myanmar women, Tun and Ostergren (2020) detected a significant association between husbands’ resort to physical violence targeting their wives and their attitude of controlling behavior targeting those women. A study about seven Central America countries (Honduras, Costa Rica, El Salvador, Nicaragua, Guatemala, Belize, Panama) detected that men who beat their partners are more likely to resort to more oppressive controlling behaviors (Velzeboer et al., 2003). In another study focused on the cases of Brazil, Ethiopia, Bangladesh, Samoa, Japan, United Republic of Tanzania, Thailand, Namibia, Peru, Serbia and Montenegro, women who experienced sexual or physical violence from their partners were substantially exposed to more controlling behavior acts compared to those women who have never experienced partner violence (Garcia-Moreno et al., 2006). In a study about the cases of China, Sri Lanka and Bangladesh, it was determined that controlling behavior possessed an association with physical violence or with both physical and sexual violence (Fulu et al., 2013). These results are consistent with the findings of the WHO (2005) which determined that intimate partners who perpetrate physical or sexual violence are more likely to inflict controlling behavior on their partners.

As women find it right for the men to beat their partners or wives, those women become more likely to be exposed to controlling behavior. At this point, Rani and Bonu (2009), in their research conducted in seven countries (Türkiye, India, Kazakhstan, Armenia, Bangladesh, Nepal and Cambodia), found that the second highest rate of justification of wife beating among women was observed in Türkiye (49%), after India (57%). Such an approach of women toward male violence mostly stems from traditional societal norms that authorize men to perpetrate physical punishment on their partners or wives and the cultural context in which the women live (Antai, 2011). By such a justification of male violence, a woman grants her intimate partner authorisation for further violence and full control over herself. The study focusing on the case of India’s city of Delhi reached a similar result by indicating that women’s justification of violence is positively associated with husbands’ controlling behavior toward them (Mukherjee and Joshi, 2021). Similarly, Mondal and Paul (2021) found a correlation between the justification of wife beating and the controlling behavior of the male partner and the risk of being exposed to IPV in the Indian context.

According to the findings of the current research, women who experienced emotional violence are more likely to be exposed to controlling behavior. In their research focusing on the case of Myanmar, Tun and Ostergren (2020) found that lifetime emotional violence by men targeting women could be associated with husbands’ controlling behavior. The fact that exposure to emotional violence from someone other than the intimate partner increases women’s possibility to be exposed to controlling behavior of her intimate partner might stem from society’s collective justification of emotional violence targeting women. In many societies, men’s honor is associated with his capacity to control the behaviors of his partner or wife (Velzeboer et al., 2003). In a study conducted in Türkiye, exposure to controlling behaviors of their intimate partners was found to be higher for women who were exposed to emotional violence by their families of birth (Akar et al., 2010). Although not specific as emotional violence, the study conducted in Spain by Aizpurua et al. (2021) also detected a positive correlation between exposure to violence by someone outside the family and exposure to controlling behavior.

The findings revealing that being afraid of the intimate partner increases the possibility of exposure to controlling behavior may indicate that men use fear as a supportive instrument for dominating women, and by this way, they deepen the impact of their controlling behaviors over women. Fear from the partner in a relationship may stem from perpetrator partners’ behaviors that emerged in the form of threats or bodily injuries with the aim of exerting power or having control over them in the past (Waila et al., 2022). Fear in a relationship may also stem from the possible collapse of “the myth of their fine family life,” when women take a counter-action against their intimate partners (Gelles, 1976). Fear will thus lead to a reluctance to challenge controlling behavior for the sake of family life. Significant studies detected a correlation between women’s fear from their intimate partners in a relationship and their experience of violence (Bradley et al., 2002; Paul et al., 2006; Kwagala et al., 2013).

Several determinants of controlling behavior detected in this research such as living in rural areas and therefore being affected from the patriarchal culture as well as strict social control prevailing in rural areas, being afraid of the intimate partner, being exposure to emotional violence from the intimate partner or from someone other than the intimate partner are directly related with the vulnerability and therefore autonomy of women. Women’s lack of autonomy in their relationships may lead to low self-efficacy, which increases their likelihood of experiencing domestic violence (Mondal and Paul, 2021). Self-efficacy, which was defined by Bandura (1997) as ‘the beliefs in one’s capabilities to organize and execute the courses of action required to produce given attainments’, can be regarded as a counter-tactic to resist the controlling behavior of male partner. Self-evaluation of one’s self-efficacy affects the goals she strives for, the amount of energy she expends to achieve her goals and her behavioral performance (Forsyth and Carey, 1998).

Considering women’s decision-making autonomy and assertiveness on issues regarding their sexual health, for example, sexual controlling behaviors, such as involuntary sexual intercourse and unsafe sex, cause problems for women’s gynacological health that are at least as serious as sexual assault (Campbell et al., 2002). When women have accurate knowledge about HIV/AIDS and prevention, do not perceive barriers to safe sex and, more importantly, believe in their ability to negotiate condom use (condom negotiation self-efficacy) to resist men’s controlling behavior, they are more likely to be protected from HIV/AIDS (Patrão et al., 2021). In this sense, it was determined that condom use is positively correlated with condom negotiation self-efficacy (Patrão and McIntyre, 2018). Women’s inability or fear of negotiating safer sex and confronting confrontational partners is an important issue in determining women’s beliefs about their self-efficacy (Patrão et al., 2021). Women’s feeling of self-efficacy depends on fewer immediate threats (which is directly related to women’s exposure to IPV and controlling behaviors) and more room to negotiate their partner’s condom use. In the case of conflict between partners (e.g., disagreements or IPV), women may refrain from insisting and prefer to comply with their husband’s wishes. In this case, women’s perception of risk and how it relates to their decision-making becomes important (Mpondo et al., 2015). In the African context, for example, many Mozambican women are not able to create a healthy communication environment that can facilitate negotiation due to gender norms embedded in society, and their negotiation self-efficacy levels regarding condom use remain low (Patrão and McIntyre, 2017). It can therefore be said that men’s controlling behavior, often backed up by physical and sexual violence, directly determines women’s position in the decision-making processes in a relationship. From this perspective, both women’s autonomy and their beliefs about their self-efficacy depend on their capacity to resist the controlling behavior of their male partners.

At the end, one’s application of any type of IPV (whether it is physical or non-physical, including controlling behavior) in an intimate relationship reveals the nature of power and the dynamics of control in wider context. As men use violence as a tactic for possessing an exact control over their partner, a more general strategy for power is reproduced in societies (Johnson, 2010). The tactics to control the partner in a relationship is similar to those controlling tactics used for reproducing racism, anti-Semitism, heterosexism, ageism and other instances of group dominance (Pence et al., 1993). Therefore, controlling behavior at the micro level can be considered as the starting point of power and domination relations at the macro level.

A significant manifestation of male controlling behavior over women at the macro level is hegemonic masculinity which was clarified by Connell (2005) as ‘the configuration of gender practice which embodies the currently accepted answer to the problem of the legitimacy of patriarchy, which guarantees (or is taken to guarantee) the dominant position of men and the subordination of women’. Thereby, masculinity cannot be regarded as a fixed and unchanging entity that is embedded in individuals’ personality traits or body. Masculinities are practical configurations performed in social action and thusly may vary based on gender relations in different social settings (Connell and Messerschmidt, 2005). Controlling behaviors of men perpetually reproduce masculinity in each social setting around the world. The boundaries of masculinity are drawn as far as the social setting allows.

Women’s options of social action in a social setting are determined by the extent and severity of controlling behavior targeting them. As Gramsci (1971) regarded each person as a potential philosopher in society outside his/her professional work life by contributing to the existence of new ways of thinking, consciously preferring a particular moral conduct and participating in the creation, sustainment and modification of a specific conception of the world. For him, however, each person cannot perform this function in society, because of structural hegemonic obstacles embedded in different social settings. Thus, gender-based hegemonic character of controlling behavior mainly stems from its isolating effect restricting women in daily life and public space. These elements are crucial elements in terms of the construction of hegemonic masculinity in societies.

## 6. Conclusion

To our knowledge, our current study was the first to examine the relationship between various risk factors and controlling behavior in a nationally representative sample in Türkiye. Intimate partner controlling behavior is a form of IPV that needs to be addressed in detail in order to dismantle the power relations embedded in male-dominated social structures and to ensure gender equality in societies. What makes controlling behavior toward the unmarried partner or wife important is that a relationship or a family is the place where gender roles are distributed at first hand, and controlling behavior by the intimate partner reproduces social power relations at the micro level within the family or relationship. Therefore, the main purpose of this study was to investigate the basic socio-demographic, economic and violence-related determinants of controlling behavior and in this way make a modest contribution to challenging the structural (institutional, ideological and discursive) underpinnings of male-dominated society.

In the light of the findings of the study, public policies are needed to nullify the effects of controlling behavior toward women on male domination and patriarchy in societies, to raise public awareness of the issue and to provide women with the mechanisms and means to resist male controlling behavior. The economic independence of women should be guaranteed against the overbearing authority of men, which is a product of men’s material power caused by the unequal distribution of wealth between the genders. A legal and institutional basis for gender equality that prioritizes women’s position as active subjects of society should be established. Taking into account the impact of other forms of violence (economic, physical, sexual and emotional or psychological) on controlling behavior, a holistic approach should be developed to prevent all forms of violence. Institutions aiming to minimize gender-based violence and controlling behavior should take stable steps based on more robust decisions (Alkan et al., 2021). Curriculum at schools should be redesigned to ensure that attitudes of controlling behavior are condemned, and the discursive basis of patriarchy is challenged. Educational programs and communication instruments should be introduced to guide women about their rights and their options of legal action when faced with controlling behavior.

Based on the findings of this study about the main determinants of controlling behavior, further studies are needed to examine women’s tactics of resistance to men’s controlling behavior in Türkiye. Such studies should focus on how women attempt to construct a counter-hegemony that can challenge the structural hegemony of masculinity and patriarchy embedded within society and traditions.

## Data availability statement

The datasets presented in this article are not readily available because the data underlying this study is subject to third-party restrictions by the Turkey Statistical Institute. Data are available from the Turkish Statistical Institute (bilgi@tuik.gov.tr) for researchers who meet the criteria for access to confidential data. The authors of the study did not receive any special privileges in accessing the data. Requests to access the datasets should be directed to bilgi@tuik.gov.tr.

## Ethics statement

Ethical review and approval was not required for the study on human participants in accordance with the local legislation and institutional requirements. Written informed consent from the patients/ participants or patients/participants' legal guardian/next of kin was not required to participate in this study in accordance with the national legislation and the institutional requirements.

## Author contributions

ÖA conceived and led the design and development of the study proposal. ÖA and BB supervised the data collection, led the data analysis, and drafted the manuscript. BB made substantial contributions to the conceptualization and design of the study, data interpretations, and writing the manuscript. All authors read and approved the final version of the manuscript.

## Conflict of interest

Author ÖA was employed by Master Araştırma Eğitim ve Danışmanlık Hizmetleri Ltd. Şti.

The remaining author declares that the research was conducted in the absence of any commercial or financial relationships that could be construed as a potential conflict of interest.

## Publisher’s note

All claims expressed in this article are solely those of the authors and do not necessarily represent those of their affiliated organizations, or those of the publisher, the editors and the reviewers. Any product that may be evaluated in this article, or claim that may be made by its manufacturer, is not guaranteed or endorsed by the publisher.

## References

[ref1] ABS. (2009). Conceptual framework for family and domestic violence. Available at: https://www.abs.gov.au/ausstats/abs@.nsf/Products/F346821A88ED5F6ACA2575B700176310 (Accessed January 01, 2023).

[ref2] Ackerson L. K. Kawachi I. Barbeau E. M. Subramanian S. (2008). Effects of individual and proximate educational context on intimate partner violence: a population-based study of women in India. Am. J. Public Health 98, 507–514. doi: 10.2105/AJPH.2007.113738, PMID: 18235066 PMC2253590

[ref3] Aizer A. (2010). The gender wage gap and domestic violence. Am. Econ. Rev. 100, 1847–1859. doi: 10.1257/aer.100.4.1847, PMID: 25110354 PMC4123456

[ref4] Aizpurua E. Copp J. Ricarte J. J. Vázquez D. (2021). Controlling behaviors and intimate partner violence among women in Spain: an examination of individual, partner, and relationship risk factors for physical and psychological abuse. J. Interpers. Violence 36, 231–254. doi: 10.1177/0886260517723744, PMID: 29294888

[ref5] Akar T. Aksakal F. N. Demirel B. Durukan E. Özkan S. (2010). The prevalence of domestic violence against women among a group woman: Ankara, Turkey. J. Family Violence 25, 449–460. doi: 10.1007/s10896-010-9306-8

[ref6] Algül Y. Yarbaşı İ. Y. (2021). The effects of employment and financial empowerment of women on domestic violence: the case of Turkey. Erciyes Univ. J. Faculty Econ. Admin. Sci., 197–220. doi: 10.18070/erciyesiibd.935635

[ref7] Ali T. S. Abbas A. Ather F. (2014). Associations of controlling behavior, physical and sexual violence with health symptoms. J. Women's Health Care 3, 1–6. doi: 10.4172/2167-0420.1000202

[ref8] Alkan Ö. Özar Ş. Ünver Ş. (2021). Economic violence against women: a case in Turkey. PLoS One 16:e0248630. doi: 10.1371/journal.pone.0248630, PMID: 33720990 PMC7959383

[ref9] Alkan Ö. Serçemeli C. Özmen K. (2022). Verbal and psychological violence against women in Turkey and its determinants. PLoS One 17:e0275950. doi: 10.1371/journal.pone.0275950, PMID: 36215284 PMC9550074

[ref10] Alkan Ö. Tekmanlı H. H. (2021). Determination of the factors affecting sexual violence against women in Turkey: a population-based analysis. BMC Womens Health 21, 1–15. doi: 10.1186/s12905-021-01333-1 33952220 PMC8097900

[ref11] Antai D. (2011). Controlling behavior, power relations within intimate relationships and intimate partner physical and sexual violence against women in Nigeria. BMC Public Health 11, 1–11. doi: 10.1186/1471-2458-11-511 21714854 PMC3161889

[ref12] Åsling-Monemi K. Tabassum Naved R. Persson L. Å. (2008). Violence against women and the risk of under-five mortality: analysis of community-based data from rural Bangladesh. Acta Paediatr. 97, 226–232. doi: 10.1111/j.1651-2227.2007.00597.x, PMID: 18254912

[ref13] Babu B. V. Kar S. K. (2009). Domestic violence against women in eastern India: a population-based study on prevalence and related issues. BMC Public Health 9, 1–16. doi: 10.1186/1471-2458-9-129 19426515 PMC2685379

[ref14] Baker C. K. Carreño P. K. (2016). Understanding the role of technology in adolescent dating and dating violence. J. Child Fam. Stud. 25, 308–320. doi: 10.1007/s10826-015-0196-5

[ref15] Bandura A. (1997). Self-efficacy: the exercise of control. New York: W. H. Freeman.

[ref16] Batinic B. Duisin D. Barisic J. (2013). Obsessive versus delusional jealousy. Psychiatr. Danub. 25:339:334. PMID: 24048408

[ref17] Belotti F. Ieracitano F. Donato S. Comunello F. (2022). Towards ‘romantic media ideologies’: digital dating abuse seen through the lens of social media and/or dating in teenage narratives. Commun. Rev. 25, 30–53. doi: 10.1080/10714421.2022.2033576

[ref18] Bobonis G. J. González-Brenes M. Castro R. (2013). Public transfers and domestic violence: the roles of private information and spousal control. Am. Econ. J. Econ. Pol. 5, 179–205. doi: 10.1257/pol.5.1.179

[ref19] Bradley F. Smith M. Long J. O'Dowd T. (2002). Reported frequency of domestic violence: cross sectional survey of women attending general practice. BMJ 324:271. doi: 10.1136/bmj.324.7332.271, PMID: 11823359 PMC65059

[ref20] Campbell J. Jones A. S. Dienemann J. Kub J. Schollenberger J. O'Campo P.. (2002). Intimate partner violence and physical health consequences. Arch. Intern. Med. 162, 1157–1163. doi: 10.1001/archinte.162.10.1157 12020187

[ref21] CEDAW Committee. (1992). General Recommendation No. 19: Violence against women. Available at: https://www.refworld.org/docid/52d920c54.html (Accessed January 05, 2023).

[ref22] Chacham A. S. Simão A. B. Caetano A. J. (2016). Gender-based violence and sexual and reproductive health among low-income youth in three Brazilian cities. Reprod. Health Matters 24, 141–152. doi: 10.1016/j.rhm.2016.06.009, PMID: 27578347

[ref23] Connell R. (2005). Masculinities (2 ed.). University of California Press.

[ref24] Connell R. W. Messerschmidt J. W. (2005). Hegemonic masculinity: rethinking the concept. Gend. Soc. 19, 829–859. doi: 10.1177/0891243205278639

[ref25] CPS. (2017). Controlling or Coercive Behaviour in an Intimate or Family Relationship. Available at: https://www.cps.gov.uk/legal-guidance/controlling-or-coercive-behaviour-intimate-or-family-relationship (Accessed January 10, 2023).

[ref26] Daspe M.-È. Vaillancourt-Morel M.-P. Lussier Y. Sabourin S. (2018). Facebook use, Facebook jealousy, and intimate partner violence perpetration. Cyberpsychol. Behav. Soc. Netw. 21, 549–555. doi: 10.1089/cyber.2018.0159, PMID: 30212246

[ref27] Devries K. M. Mak J. Y. Garcia-Moreno C. Petzold M. Child J. C. Falder G.. (2013). The global prevalence of intimate partner violence against women. Science 340, 1527–1528. doi: 10.1126/science.1240937 23788730

[ref28] DGSW. (2009). National Research on Domestic Violence against Women in Turkey. Available at: https://hips.hacettepe.edu.tr/en/analysis_and_report-320 (Accessed September 14, 2022).

[ref29] DGSW. (2015). National Research on Domestic Violence against Women in Turkey. Available at: http://www.hips.hacettepe.edu.tr/ING_SUMMARY_REPORT_VAW_2014.pdf (Accessed September 14, 2022).

[ref30] Durevall D. Lindskog A. (2015). Intimate partner violence and HIV in ten sub-Saharan African countries: what do the demographic and health surveys tell us? Lancet Glob. Health 3, e34–e43. doi: 10.1016/S2214-109X(14)70343-2, PMID: 25539967

[ref31] Erman T. (1998). The impact of migration on Turkish rural women: four emergent patterns. Gend. Soc. 12, 146–167. doi: 10.1177/089124398012002003

[ref32] Erman T. (2001). Rural migrants and patriarchy in Turkish cities. Int. J. Urban Reg. Res. 25, 118–133. doi: 10.1111/1468-2427.00301

[ref33] Erten B. Keskin P. (2018). For better or for worse?: education and the prevalence of domestic violence in Turkey. Am. Econ. J. Appl. Econ. 10, 64–105. doi: 10.1257/app.20160278

[ref34] Fanslow J. L. Malihi Z. A. Hashemi L. Gulliver P. J. McIntosh T. K. (2021). Lifetime prevalence of intimate partner violence and disability: results from a population-based study in New Zealand. Am. J. Prev. Med. 61, 320–328. doi: 10.1016/j.amepre.2021.02.022, PMID: 34419229

[ref35] Farmer A. Tiefenthaler J. (1996). Domestic violence: the value of services as signals. Am. Econ. Rev. 86, 274–279.

[ref36] Fawson P. R. (2015). Controlling behaviors as a predictor of partner violence among heterosexual female and male adolescents. Partn. Abus. 6, 217–229. doi: 10.1891/1946-6560.6.2.217

[ref37] Forsyth A. D. Carey M. P. (1998). Measuring self-efficacy in the context of HIV risk reduction: research challenges and recommendations. Health Psychol. 17, 559–568. doi: 10.1037/0278-6133.17.6.559, PMID: 9848807

[ref38] Fulu E. Jewkes R. Roselli T. Garcia-Moreno C. (2013). Prevalence of and factors associated with male perpetration of intimate partner violence: findings from the UN multi-country cross-sectional study on men and violence in Asia and the Pacific. Lancet Glob. Health 1, e187–e207. doi: 10.1016/S2214-109X(13)70074-3, PMID: 25104345

[ref39] Garcia-Moreno C. Jansen H. A. Ellsberg M. Heise L. Watts C. H. (2006). Prevalence of intimate partner violence: findings from the WHO multi-country study on women's health and domestic violence. Lancet 368, 1260–1269. doi: 10.1016/S0140-6736(06)69523-8 17027732

[ref40] Gelles R. J. (1976). Abused wives: why do they stay. J. Marriage Fam. 38, 659–668. doi: 10.2307/350685

[ref41] Gilchrist G. Canfield M. Radcliffe P. d'Oliveira A. F. P. L. (2017). Controlling behaviours and technology-facilitated abuse perpetrated by men receiving substance use treatment in England and Brazil: prevalence and risk factors. Drug Alcohol Rev. 36, 52–63. doi: 10.1111/dar.12521, PMID: 28134494 PMC5299471

[ref42] Goode W. J. (1971). Force and violence in the family. J. Marriage Fam. 33, 624–636. doi: 10.2307/349435

[ref43] Graham-Kevan N. Archer J. (2003). Intimate terrorism and common couple violence: a test of Johnson's predictions in four British samples. J. Interpers. Violence 18, 1247–1270. doi: 10.1177/0886260503256656, PMID: 19774764

[ref44] Graham-Kevan N. Archer J. (2008). Does controlling behavior predict physical aggression and violence to partners? J. Fam. Violence 23, 539–548. doi: 10.1007/s10896-008-9162-y

[ref45] Gramsci A. (1971). Selections from the Prison Notebooks of Antonio Gramsci (Q. H. G. N. S. E. Trans.). eds. Lawrence and Wishart, London.

[ref46] Hidrobo M. Fernald L. (2013). Cash transfers and domestic violence. J. Health Econ. 32, 304–319. doi: 10.1016/j.jhealeco.2012.11.002, PMID: 23237793

[ref47] Hines D. A. Brown J. Dunning E. (2007). Characteristics of callers to the domestic abuse helpline for men. J. Fam. Violence 22, 63–72. doi: 10.1007/s10896-006-9052-0

[ref48] Igwe C. P. Yusuf O. B. Fawole O. I. (2021). Prevalence and correlates of intimate partner violence experience among partners of naval personnel in Lagos, Nigeria. Int. Q. Community Health Educ. 42, 63–72. doi: 10.1177/0272684X20974223, PMID: 33215574

[ref49] Jewkes R. (2002). Intimate partner violence: causes and prevention. Lancet 359, 1423–1429. doi: 10.1016/S0140-6736(02)08357-5 11978358

[ref50] Jewkes R. K. Dunkle K. Nduna M. Shai N. (2010). Intimate partner violence, relationship power inequity, and incidence of HIV infection in young women in South Africa: a cohort study. Lancet 376, 41–48. doi: 10.1016/S0140-6736(10)60548-X, PMID: 20557928

[ref51] Johnson M. P. (1995). Patriarchal terrorism and common couple violence: two forms of violence against women. J. Marriage Fam. 57, 283–294. doi: 10.2307/353683

[ref52] Johnson M. P. (2006). Conflict and control: gender symmetry and asymmetry in domestic violence. Violence Against Women 12, 1003–1018. doi: 10.1177/1077801206293328 17043363

[ref53] Johnson M. P. (2010). A typology of domestic violence: Intimate terrorism, violent resistance, and situational couple violence. Lebanon: UPNE.

[ref54] Johnson M. P. Ferraro K. J. (2000). Research on domestic violence in the 1990s: making distinctions. J. Marriage Fam. 62, 948–963. doi: 10.1111/j.1741-3737.2000.00948.x

[ref55] Kanougiya S. Sivakami M. Rai S. (2021). Predictors of spousal coercive control and its association with intimate partner violence evidence from National Family Health Survey-4 (2015-2016) India. BMC Public Health 21, 1–13. doi: 10.1186/s12889-021-12232-3 34844591 PMC8628403

[ref56] Kelly J. B. Johnson M. P. (2008). Differentiation among types of intimate partner violence: research update and implications for interventions. Fam. Court. Rev. 46, 476–499. doi: 10.1111/j.1744-1617.2008.00215.x

[ref57] Kishor S. Johnson K. (2005). Profiling domestic violence: a multi-country study. Stud. Fam. Plan. 36, 259–261.

[ref58] Krantz G. Vung N. D. (2009). 2The role of controlling behaviour in intimate partner violence and its health effects: a population based study from rural Vietnam. BMC Public Health 9, 1–11. doi: 10.1186/1471-2458-9-143 19442288 PMC2689202

[ref59] Krug E. G. Mercy J. A. Dahlberg L. L. Zwi A. B. (2002). The world report on violence and health. Lancet 360, 1083–1088. doi: 10.1016/S0140-6736(02)11133-0 12384003

[ref60] Kundapur R. Shetty S. M. Kempaller V. J. Kumar A. Anurupa M. (2017). Violence against educated women by intimate partners in urban Karnataka, India. Indian J. Community Med. 42, 147–150. doi: 10.4103/ijcm.IJCM_41_16, PMID: 28852277 PMC5561691

[ref61] Kwagala B. Wandera S. O. Ndugga P. Kabagenyi A. (2013). Empowerment, partner’s behaviours and intimate partner physical violence among married women in Uganda. BMC Public Health 13, 1–11. doi: 10.1186/1471-2458-13-1112, PMID: 24289495 PMC4219526

[ref62] Macdonald F. (2012). Spotlight on economic abuse: A literature and policy review. North Collingwood: Good Shepherd Youth & Family Service and Kildonan Uniting Care.

[ref63] Martin S. L. Moracco K. E. Garro J. Tsui A. O. Kupper L. L. Chase J. L.. (2002). Domestic violence across generations: findings from northern India. Int. J. Epidemiol. 31, 560–572. doi: 10.1093/ije/31.3.560, PMID: 12055156

[ref64] Mayda A. S. Akkuş D. (2005). Domestic violence against 116 Turkish housewives: a field study. Women Health 40, 95–108. doi: 10.1300/J013v40n03_07, PMID: 15829448

[ref65] McClintock H. F. Evenosky S. Trego M. (2022). Severity of lifetime physical intimate partner violence and controlling behavior in sub-Saharan Africa. J. Immigr. Minor. Health 24, 1508–1516. doi: 10.1007/s10903-022-01336-6, PMID: 35122552

[ref66] Mondal D. Paul P. (2021). Associations of power relations, wife-beating attitudes, and controlling behavior of husband with domestic violence against women in India: insights from the National Family Health Survey–4. Violence Against Women 27, 2530–2551. doi: 10.1177/1077801220978794, PMID: 33393878

[ref67] Mpondo F. Ruiter R. A. van den Borne B. Reddy P. S. (2015). Self-determination and gender–power relations as predictors of condom use self-efficacy among south African women. Health Psychol. Open 2, 205510291559867–205510291559811. doi: 10.1177/2055102915598676 PMC5193249 28070366

[ref68] Mukherjee R. Joshi R. K. (2021). Controlling behavior and intimate partner violence: a cross-sectional study in an urban area of Delhi, India. J. Interpers. Violence 36:NP10831-NP10842. doi: 10.1177/0886260519876720, PMID: 31561731

[ref69] Mundodan J. M. Lamiya K. Haveri S. P. (2021). Prevalence of spousal violence among married women in a rural area in North Kerala. J. Family Med. Primary Care 10, 2845–2852. doi: 10.4103/jfmpc.jfmpc_2313_20, PMID: 34660416 PMC8483127

[ref70] Nasrullah M. Zakar R. Zakar M. Z. (2014). Child marriage and its associations with controlling behaviors and spousal violence against adolescent and young women in Pakistan. J. Adolesc. Health 55, 804–809. doi: 10.1016/j.jadohealth.2014.06.013 25123525

[ref71] NSD. (2010). Timor-Leste Demographic and Health Survey, 2009–10. Available at: https://dhsprogram.com/publications/publication-fr235-dhs-final-reports.cfm (Accessed January 10, 2023).

[ref72] Ozer M. Fidrmuc J. (2017). *Male education and domestic violence in Turkey: Evidence from a natural experiment* (CESifo Working Paper Series No. 6526, Issue).

[ref73] Patrão A. L. McIntyre T. M. (2017). Socio-demographic, marital, and psychosocial factors associated with condom use negotiation self-efficacy among Mozambican women at risk for HIV infection. Int. J. Behav. Med. 24, 846–855. doi: 10.1007/s12529-017-9681-0, PMID: 28831688

[ref74] Patrão A. L. McIntyre T. (2018). Socio-demographic, marital and psychosocial predictors of safe sex behaviour among Mozambican women at risk for HIV/AIDS. Afr. J. AIDS Res. 17, 323–331. doi: 10.2989/16085906.2018.1536672, PMID: 30466355

[ref75] Patrão A. L. McIntyre T. M. Costa E. C. Matediane E. Azevedo V. (2021). Testing the effectiveness of two psychosocial interventions on socio-cognitive risk factors for HIV/AIDS in Mozambican women: a randomized controlled trial. AIDS Educ. Prev. 33, 169–186. doi: 10.1521/aeap.2021.33.3.169, PMID: 34014113

[ref76] Paul G. Smith S. M. Long J. (2006). Experience of intimate partner violence among women and men attending general practices in Dublin, Ireland: a cross-sectional survey. Eur. J. Gen. Pract. 12, 66–69. doi: 10.1080/13814780600757344, PMID: 16945879

[ref77] Pence E. Paymar M. Ritmeester T. (1993). Education groups for men who batter: the Duluth model. New York: Springer Publishing Company.

[ref78] Plichta S. B. (2004). Intimate partner violence and physical health consequences: policy and practice implications. J. Interpers. Violence 19, 1296–1323. doi: 10.1177/0886260504269685 15534333

[ref79] Rani M. Bonu S. (2009). Attitudes toward wife beating: a cross-country study in Asia. J. Interpers. Violence 24, 1371–1397. doi: 10.1177/0886260508322182 18718881

[ref80] Ruggieri S. Bonfanti R. C. Passanisi A. Pace U. Schimmenti A. (2021). Electronic surveillance in the couple: the role of self-efficacy and commitment. Comput. Hum. Behav. 114, 106577–106510. doi: 10.1016/j.chb.2020.106577

[ref81] Solanke B. L. Kupoluyi J. A. Awoleye A. F. Adewole O. E. Babalola O. B. (2023). Women’s ability to negotiate safer sex with partners by contraceptive status among a nationally representative sample of married women in Nigeria. Contra. Reprod. Med. 8, 1–14. doi: 10.1186/s40834-023-00214-2 PMC9976491 36855163

[ref82] Stark E. (2007). Coercive control: The entrapment of women in personal life. Oxford: Oxford University Press.

[ref83] Stonard K. E. (2019). Technology-assisted adolescent dating violence and abuse: a factor analysis of the nature of electronic communication technology used across twelve types of abusive and controlling behaviour. J. Child Fam. Stud. 28, 105–115. doi: 10.1007/s10826-018-1255-5

[ref84] Straus M. A. (2008). Dominance and symmetry in partner violence by male and female university students in 32 nations. Child Youth Serv. Rev. 30, 252–275. doi: 10.1016/j.childyouth.2007.10.004

[ref85] Tanimu T. S. Yohanna S. Omeiza S. Y. (2016). The pattern and correlates of intimate partner violence among women in Kano, Nigeria. African J. Primary Health Care Family Med. 8, 1–6. doi: 10.4102/phcfm.v8i1.1209 PMC5153410 28155317

[ref86] Trevillion K. Oram S. Feder G. Howard L. M. (2012). Experiences of domestic violence and mental disorders: a systematic review and meta-analysis. PLoS One 7:e51740. doi: 10.1371/journal.pone.0051740, PMID: 23300562 PMC3530507

[ref87] Tun T. Ostergren P.-O. (2020). Spousal violence against women and its association with sociodemographic factors and husbands’ controlling behaviour: the findings of Myanmar demographic and health survey (2015–2016). Glob. Health Action 13:1844975. doi: 10.1080/16549716.2020.1844975, PMID: 33215577 PMC7737679

[ref88] UN. (1993). Vienna Declaration and Programme of Action. Available at: https://www.ohchr.org/en/instruments-mechanisms/instruments/vienna-declaration-and-programme-action (Accessed January 05, 2023).

[ref89] UNAIDS. (2011). UNAIDS 2011 world AIDS day report. Geneva: Joint United Nations Programme on HIV/AIDS.

[ref90] UNGA. (1979). Convention on the Elimination of All Forms of Discrimination against Women. Available at: https://www.mfa.gov.tr/convention-on-the-elimination-of-all-forms-of-discrimination-against-women.en.mfa (Accessed January 03, 2023).

[ref91] UNODC. (2021). Killings of women and girls by their intimate partner or other family members: Global estimates 2020. Available at: https://reliefweb.int/report/world/killings-women-and-girls-their-intimate-partner-or-other-family-members-global (Accessed January 15, 2023).

[ref92] UNWOMEN. (2020). The economic and social impact of COVID-19 on women and men: Rapid Gender Assessment of COVID-19 implications in Turkey. Available at: https://eca.unwomen.org/en/digital-library/publications/2020/06/the-impact-of-covid19-on-women-and-men-rapid-gender-assessment-of-covid19-implications-in-turkey (Accessed January 17, 2023).

[ref93] Velzeboer M. Ellsberg M. Arcas C. C. García-Moreno C. (2003). Violence against women: The health sector responds. Pan American Health Organization, Pan American Sanitary Bureau.

[ref94] Waila J. Lule H. Lowery Wilson M. Bärnighausen T. Abio A. (2022). Ugandan men exposed to intimate partner violence: a cross-sectional survey of nationally representative data. J. Prevent. 43, 567–588. doi: 10.1007/s10935-022-00683-2, PMID: 35650366 PMC9252969

[ref95] Walker K. Sleath E. Tramontano C. (2021). The prevalence and typologies of controlling behaviors in a general population sample. J. Interpers. Violence 36:NP474-NP503. doi: 10.1177/0886260517731785, PMID: 29294941

[ref96] Watts C. Zimmerman C. (2002). Violence against women: global scope and magnitude. Lancet 359, 1232–1237. doi: 10.1016/S0140-6736(02)08221-1, PMID: 11955557

[ref97] WEF. (2022). Global Gender Gap Report 2022. Available at: https://www3.weforum.org/docs/WEF_GGGR_2022.pdf (Accessed January 16, 2023).

[ref98] WHO. (2005). WHO multi-country study on women's health and domestic violence against women: initial results on prevalence, health outcomes and women's responses. World health Organization. Available at: https://apps.who.int/iris/handle/10665/43309 (Accessed January 12, 2023).

[ref99] WHO. (2021). Violence against women prevalence estimates, 2018: global, regional and national prevalence estimates for intimate partner violence against women and global and regional prevalence estimates for non-partner sexual violence against women. Available at: https://www.who.int/publications/i/item/9789240022256 (Accessed December 21, 2022). 10.1016/S0140-6736(21)02664-7 PMC8885817 35182472

[ref100] WHO and PAHO. (2012). Understanding and addressing violence against women: Intimate partner violence. Available at: https://apps.who.int/iris/handle/10665/77432 (Accessed December 30, 2022).

[ref101] WHO and UNAIDS. (2010). Addressing violence against women and HIV/AIDS: What works? Available at: https://apps.who.int/iris/handle/10665/44378 (Accessed December 30, 2022).

[ref102] Williams L. (2012). ““Love is…”: how adolescents define and experience romantic love” in The psychology of love. ed. Paludi M. A., vol. 3, 1–20.

